# Enhanced Immunological Recovery With Early Start of Antiretroviral Therapy During Acute or Early HIV Infection–Results of Italian Network of ACuTe HIV InfectiON (INACTION) Retrospective Study

**DOI:** 10.20411/pai.v5i1.341

**Published:** 2020-02-24

**Authors:** Antonio Muscatello, Silvia Nozza, Massimiliano Fabbiani, Ilaria De Benedetto, Marco Ripa, Raffaele Dell'acqua, Andrea Antinori, Carmela Pinnetti, Andrea Calcagno, Micol Ferrara, Emanuele Focà, Eugenia Quiros-Roldan, Diego Ripamonti, Marco Campus, Benedetto Maurizio Celesia, Carlo Torti, Lucio Cosco, Antonio Di Biagio, Stefano Rusconi, Giulia Marchetti, Cristina Mussini, Roberto Gulminetti, Antonella Cingolani, Gabriella D'ettorre, Giordano Madeddu, Antonina Franco, Giancarlo Orofino, Nicola Squillace, Andrea Gori, Giuseppe Tambussi, Alessandra Bandera, On Behalf Of Inaction Study Group.

**Affiliations:** 1 Infectious Diseases Unit; Department of Internal Medicine; IRCCS Ca' Granda Foundation Maggiore Hospital; Milan, Italy; 2 Clinic of Infectious Diseases; San Raffaele Hospital; University Vita Salute; Milan, Italy; 3 Infectious and Tropical Diseases Unit; Azienda Ospedaliero-Universitaria Senese; Siena, Italy; 4 Department of Medical Sciences; Unit of Infectious Diseases; University of Turin; Amedeo di Savoia Hospital; Turin, Italy; 5 National Institute for Infectious Diseases Lazzaro Spallanzani IRCCS; Rome, Italy; 6 Division of Infectious and Tropical Diseases; University of Brescia; ASST Spedali Civili Hospital; Brescia, Italy; 7 Infectious Disease Unit; ASST Papa Giovanni XXIII; Bergamo, Italy; 8 Infectious Diseases Unit; SS Trinità Hospital; ASSL Cagliari, Italy; 9 Unit of Infectious Diseases; Garibaldi Hospital; Catania, Italy; 10 Unit of Infectious Diseases; Department of Medical and Surgical Sciences; University “Magna Graecia;” Catanzaro, Italy; 11 Infectious Diseases Unit; “Pugliese-Ciaccio” Hospital; Catanzaro, Italy; 12 Department of Infectious Diseases; IRCCS AOU San Martino IST; (DISSAL); University of Genoa; Genoa, Italy; 13 Infectious Diseases Unit; Department of Biomedical and Clinical Sciences “Luigi Sacco” Hospital; University of Milan, Italy; 14 Clinic of Infectious Diseases; Department of Health Sciences; University of Milan; ASST Santi Paolo e Carlo; Milan, Italy; 15 Clinic of Infectious Diseases; University of Modena and Reggio Emilia; Modena Hospital; Italy; 16 Institute of Clinical Infectious Diseases; Agostino Gemelli Hospital; Catholic University of Sacred Heart; Rome, Italy; 17 Infectious Diseases Unit; Umberto I Hospital; La Sapienza University; Rome, Italy; 18 Department of Clinical and Experimental Medicine; Unit of Infectious Diseases; University of Sassari, Italy; 19 Infectious Diseases Unit; ASP Siracusa, Italy; 20 Unit of Infectious Diseases; Divisione A; Amedeo di Savoia Hospital; Turin, Italy; 21 Infectious Diseases Unit; Department of Internal Medicine; ASST San Gerardo; Monza, Italy, University of Milano-Bicocca; Milan, Italy; 22 School of Medicine and Surger; University of Milan, Italy

**Keywords:** acute/early HIV infection, early antiretroviral treatment, optimal immunological recovery, intensified antiretroviral regimen

## Abstract

**Background:**

Viral load peak and immune activation occur shortly after exposure during acute or early HIV infection (AEHI). We aimed to define the benefit of early start of antiretroviral treatment (ART) during AEHI in terms of immunological recovery, virological suppression, and treatment discontinuation.

**Setting:**

Patients diagnosed with AEHI (Fiebig stages I-V) during 2008-2014 from an analysis of 20 Italian centers.

**Methods:**

This was an observational, retrospective, and multicenter study. We investigated the effect of early ART (defined as initiation within 3 months from AEHI diagnosis) on time to virological suppression, optimal immunological recovery (defined as CD4 count ≥500/µL, CD4 ≥30%, and CD4/CD8 ≥1), and first-line ART regimen discontinuation by Cox regression analysis.

**Results:**

There were 321 patients with AEHI included in the study (82.9% in Fiebig stage III-V). At diagnosis, the median viral load was 5.67 log_10_ copies/mL and the median CD4 count was 456 cells/µL. Overall, 70.6% of patients started early ART (median time from HIV diagnosis to ART initiation 12 days, IQR 6-27). Higher baseline viral load and AEHI diagnosis during 2012-2014 were independently associated with early ART. HBV co-infection, baseline CD4/CD8 ≥1, lower baseline HIV-RNA, and AEHI diagnosis in recent years (2012-2014) were independently associated with a shorter time to virological suppression. Early ART emerged as an independent predictor of optimal immunological recovery after adjustment for baseline CD4 (absolute and percentage count) and CD4/CD8 ratio. The only independent predictor of first-line ART discontinuation was an initial ART regimen including > 3 drugs.

**Conclusions:**

In a large cohort of well-characterized patients with AEHI, we confirmed the beneficial role of early ART on CD4+ T-cell recovery and on rates of CD4/CD8 ratio normalization. Moreover, we recognized baseline CD4/CD8 ratio as an independent factor influencing time to virological response in the setting of AEHI, thus giving new insights into research of immunological markers associated with virological control.

## INTRODUCTION

Acute or early HIV infection (AEHI) is a brief, rarely diagnosed, but critical phase of HIV infection. AEHI is known to be characterized by very high levels of HIV-RNA load and several studies have shown that HIV transmission is greatly amplified during AEHI, thus defining a relevant contribution of this phase to onward HIV transmission [1, 2]. During AEHI, long-lived reservoirs of virus are established early on, representing a major obstacle to achieving HIV eradication [3, 4]. Moreover, immune dysregulation is rapidly detected after viral infection contributing to over-heightened immune activation [5, 6].

Several studies assessing antiretroviral treatment (ART) during AEHI have suggested beneficial effects on biological markers of disease progression, such as reduction of viremia [7], lower viral set point [8], lower probability of transmission [9, 10], and a reduced number of infected cells limiting the size of the latent pool of HIV-1-infected CD4+ T cells [11, 12]. Moreover, it has been shown that starting ART in the initial phase of infection might allow for the preservation of the immune system, and specifically of: (1) the CD4+ T-cell count and CD4/CD8 ratio [13-15]; (2) HIV-specific immune responses, preserving the ability to control viral replication [16]; (3) higher levels of mucosal and systemic Th17 cells; (4) lower levels of B-lymphocyte activation, and (5) reconstitution of the NK phenotype [12, 17, 18]. Despite the fact that evidence from multiple studies strongly supports the benefit of early ART in AEHI [19-20], the optimal ART strategy to achieve rapid viral suppression, optimal immune recovery, and longer durability of regimens needs to be clarified.

Thus, we used retrospective data from a large national cohort to assess whether an early start of ART during AEHI is associated with an optimal immunological recovery, the timing of virological suppression, and the risk of discontinuation of first-line antiretroviral regimen.

## PATIENTS AND METHODS

### Study Design

This was a retrospective, observational, multicenter cohort study including patients with AEHI diagnosed between January 1, 2008 and December 31, 2014 at 20 Italian centers included in a national network (INACTION - Italian Network on AcuTe HIV InfectiON).

### Inclusion Criteria

Cases of AEHI were identified by a retrospective analysis of clinical files and databases of each participating center. Patients were included in the study if they presented at least 1 criterion for HIV infection diagnosis (A) and concomitantly at least 1 criterion for acute or early HIV infection (B). Criteria for (A) were 1) p24 reactivity, 2) HIV-RNA and/or HIV-DNA PCR detectable viral load; Criteria for (B) were 1) negative HIV-antibodies or low titer reactivity in an ELISA third (or further) generation assay, 2) less than 3 positive bands in a Western blot test including p24, gp160/gp120, or gp41, 3) less than 2 positive bands in a RIBA test including reactivity for gp41. Patients with AEHI were classified according to Fiebig criteria [21]; only patients with Fiebig stages I-V were included, while those with Fiebig stage VI or with no available Fiebig stage were excluded.

### Type and Timing of Antiretroviral Treatment (ARV)

The type of regimen (ie, the number of agents and the drug class included) and the follow-up schedule were selected by a single clinician, according to national or local guidelines. Patients who started any ART regimen within 3 months of the AEHI diagnosis were classified as early ART group (Early ART), while the other patients were included in the late ART group (Late ART).

### Endpoints and Definitions

The primary endpoint was the optimal immune recovery defined as a composite endpoint including the first occurrence of CD4 count ≥ 500/µL, CD4/CD8 ratio ≥1, and CD4 ≥ 30% after ART initiation [22].

The secondary endpoints were the rate of virological suppression and the risk of discontinuation of a first-line antiretroviral regimen. Virological suppression was defined as reaching HIV-RNA < 50 copies/mL. Discontinuation of first-line antiretroviral regimen was defined as the change of any drug in the regimen, new drugs added to the regimen, or ending the regimen.

### Data Collecting

Demographical (age at diagnosis, gender), epidemiological (mode of HIV transmission, date of last negative/undetermined HIV test, date of first reactive HIV test), clinical (symptoms at presentation, CDC stage, comorbidities), and laboratory (plasma HIV-RNA load, CD4 and CD8 T-cell count) data were assessed at the time of AEHI diagnosis. Follow-up was carried out until June 30, 2016 or the last available visit. During follow-up, we assessed viro-immunological parameters, clinical or laboratory adverse events, and hospital admissions or death occurring between AEHI diagnosis and the end of follow-up. The first-line and subsequent ART regimens were recorded since AEHI diagnosis (baseline, BL) occurred until December 31, 2014.

### Ethical Approval

The study was approved by the Ethics Committee (EC) of Monza-Brianza for San Gerardo Hospital (coordinating center) in June 2014 and then by the EC of each participating center.

### Statistical Analysis

Categorical variables were described as absolute and percentage frequency; continuous variables with normal distribution were described as mean and standard deviation (SD), whereas those with non-normal distribution were described as median and interquartile range (IQR). Characteristics of Early ART and Late ART groups were compared using Student's *t* test or Mann Whitney U-test and chi-square test or Fisher test, as appropriate. Logistic regression analysis was performed to identify factors associated with early ART initiation. Incidence and predictors of time to virological suppression, time to first-line regimen discontinuation, and time to optimal immunological recovery were explored by Kaplan-Meier curves and Cox regression analysis. In all these models, variables showing a significant association with the outcome at univariate analysis were then evaluated in a multivariate model. A 2-tailed *P*-value < 0.05 was considered to be statistically significant.

All statistical analyses were performed using SPSS version 13.0 software package (SPSS Inc., Chicago, IL).

## RESULTS

### Population Characteristics

Overall, 321 patients were analyzed (Table 1). Most were males (n = 276, 86%) and acquired HIV infection through MSM intercourses (n = 184, 57.3%) at a median age of 37 years (IQR 30-46). Patients reported a median time from the last negative HIV test of 6.8 months (IQR 3.35-16.4).

**Table 1.** Population characteristics at the time of AHI diagnosis (n = 321) and comparison of Early (< 3 months from AHI diagnosis) and Late (> 3 months) ART initiation groups (n = 296).

	Total (n = 321)	Early ART (n = 209)	Late ART (n = 87)	
	N (%) or median (IQR)	N (%) or median (IQR)	N (%) or median (IQR)	*P*
**Age, years**	37 (30–46)	38 (30–46)	38 (31–46)	0.580
**Male gender**	276 (86)	181 (86.6)	75 (86.2)	1.000
**Risk factor:**				0.369
**Heterosexual**	92 (28.7)	57 (27.3)	30 (34.5)	
**Homo/bisexual**	184 (57.3)	126 (60.3)	47 (54)	
**IDU**	14 (4.4)	8 (3.8)	1 (1.1)	
**Other/unknown**	31 (9.7)	18 (8.6)	9 (10.3)	
**HBsAg+**	11 (3.4)	8 (3.8)	2 (2.3)	0.679
**HCV coinfection**	15 (4.7)	8 (3.8)	4 (4.6)	0.613
**Months from last negative HIV test**	6.8 (3.35–16.4)	7.1 (2.9–23.6)	5.9 (3.4–12)	0.065
**Year of diagnosis**				**< 0.001**
**2008–2011**	106 (33)	46 (22)	49 (56.3)	
**2012–2014**	215 (67)	163 (78)	38 (43.7)	
**Fiebig stage:**				0.370
**I**	14 (4.4)	7 (3.3)	6 (6.9)	
**II**	41 (12.8)	30 (14.4)	9 (10.3)	
**III**	54 (16.8)	38 (18.2)	12 (13.8)	
**IV**	116 (36.1)	71 (34)	36 (41.4)	
**V**	96 (29.9)	63 (30)	24 (27.6)	
**Symptomatic acute HIV infection**	227 (70.7)	158 (75.6)	52 (59.8)	**0.012**
**Fever**	177 (55.1)			
**Lymphadenopathy**	78 (24.3)			
**Rash**	55 (17.1)			
**Pharyngitis**	41 (12.8)			
**Diarrhea**	26 (8.1)			
**Headache/meningism**	15 (4.7)			
**Other**	89 (27.7)			
**CDC Stage:**				
**A**	254 (79.1)			
**B**	19 (5.9)			
**C**	5 (1.6)	3 (1.4)	1 (1.3)	0.866
**Unknown**	43 (13.4)			
**Concomitant STD**	30 (9.3)	19 (9.1)	8 (9.2)	1.000
**Baseline laboratory results:**				
**WBC, cells/μL**	5800 (4257–7345)			
**Lymphocytes, cells/μL**	2059 (1350–2900)			
**Platelets, cells/μL**	196000 (156000–229500)			
**Hemoglobin, g/dL**	14.1 (13.1–15)			
**AST, UI/L**	29 (21–47)			
**ALT, UI/L**	37 (25–63)			
**CD4, cells/μL**	456 (331–605)	435 (300–574)	478 (371–599)	**0.039**
**> 500**	122 (38)	25 (12)	3 (3.4)	**0.014**
**200–500**	144 (44.9)			
**< 200**	28 (8.7)			
**Unknown**	27 (8.4)			
**CD4%**	21.7 (14.8–30)	21 (13.8–29)	23 (16–30)	0.369
**CD4% ≥ 30%**	74 (23.1)	46 (22)	19 (21.8)	**0.025**
**CD8, cells/μL**	1031 (639.5–1833)	1027 (608–1772)	1062 (721–1715)	0.675
**CD4/CD8**	0.4 (0.22-0.7)	0.4 (0.2–0.67)	0.43 (0.26–0.80)	0.659
**CD4/CD8 ≥ 1**	30 (9.3)	19 (9.1)	7 (8)	0.257
**HIV-RNA, log10 copies/mL**	5.67 (5.00–6.36)	5.96 (5.19–6.60)	5.67 (4.99–6.36)	**< 0.001**
**ART initiation during follow up**	296 (92.2)			
**Early (< 3 months from BL) ART initiation**	209/296 (70.6)			
**First ART regimen including:**				
**NRTI**		206 (98.6)	86 (98.9)	1.000
**NNRTI**		21 (10)	38 (43.7)	**< 0.001**
**PI**		165 (78.9)	41 (47.1)	**< 0.001**
**InSTI**		93 (44.5)	11 (12.6)	**< 0.001**
**Entry inhibitor**		24 (11.5)	2 (2.3)	**0.020**
**First line ART including > 3 drugs**		81 (38.8)	4 (4.6)	**< 0.001**

**Abbreviations:** AHI Acute HIV Infection; IDU Intravenous Drug User; CDC Centers for Disease Control; WBC White Blood Cells; STD Sexually Transmitted Diseases; ART Antiretroviral Therapy, NRTI Nucleo-side Reverse Transcriptase Inhibitor; NNRTI Non-Nucleoside Reverse Transcriptase Inhibitors; PI Protease Inhibitor; InSTI Integrase Strand Transfer Inhibitor; BL Baseline.

In our population, most patients (n = 215, 67%) were diagnosed with AEHI in recent years of observation (ie, 2012-2014).

At the time of AEHI diagnosis, median plasma HIV-RNA load was 5.67 log_10_ copies/mL (IQR 5.00-6.36), with a median absolute CD4 count of 456 (IQR 331-605) cells/µL, a median percentage CD4 count of 21.7% (IQR 14.8-30), and a median CD4/CD8 ratio of 0.4 (IQR 0.22-0.7).

Symptomatic AEHI was observed in 70.7% (n = 227) of patients. Of note, an AIDS-defining event was observed in 5 (1.6%) patients at the time of AEHI diagnosis (3 patients with esophageal candidiasis and 2 with Pneumocystis jirovecii pneumonia).

At the time of AEHI diagnosis, 17.1% (n = 55) of patients were classified in Fiebig stages I-II. Patients in Fiebig stages I-II had a higher viral load (median 6.11 vs 5.63 log_10_ copies/mL, *P* = 0.001) and a higher percentage of CD4 cells (median 27% vs 21%, *P* = 0.003) when compared to Fiebig III-V, but the absolute number of CD4 was similar in the 2 groups (median 468 vs 454 cells/µL, *P* = 0.324). A higher proportion of HBsAg+ patients was observed in the Fiebig I-II group (9.1% vs 2.3%, *P* = 0.034).

### ART Initiation During Follow-Up and Comparison of Early ART and Late ART Groups

During follow-up, ART was started in 92.2% (n = 296) of patients, of whom 70.6% (n = 209) started within 3 months (Early ART) (Table 1). In the overall population, the median time from AEHI diagnosis to ART initiation was 23 days (IQR 7-148). However, it was 12 days (IQR 6-27) in the subgroup of patients who started early versus 351 (IQR 221-762) in those who started late. Nearly all patients (n = 292, 98.6%) were prescribed nucleoside reverse transcriptase inhibitors (NRTI) in their first ART regimen, associated with a protease inhibitor (PI) in most cases (n = 206, 69.6%); integrase inhibitors (InSTI) were prescribed in 104 (35.1%) patients. First-line ART consisted of more than 3 drugs in 28.7% (n = 85) of patients.

A comparison of characteristics of patients with Early ART or Late ART is represented in Table 1. Patients in the Early ART group more frequently had symptomatic AEHI (75.6% [n = 158] vs 59.8% [n = 52], *P* = 0.012) and had lower CD4+ T-cell counts (median 435 [IQR 300-574] vs 478 [371–599] cells/µL, *P* = 0.039) as well as higher baseline HIV-RNA (median 5.96 [IQR 5.19-6.60] vs 5.67 [IQR 4.99-6.36] log10 copies/mL, *P* < 0.001). Patients diagnosed after 2012 were more likely to start ART early (78% [n = 163] vs 43.7% [n = 38] than in the previous period, *P* < 0.001); moreover, in the Early ART group, regimens including more than 3 drugs were prescribed more frequently (38.8% [n = 81] vs 4.6% [n = 4], *P* < 0.001). In addition, the preferred antiretroviral regimen was PI-based (78.9% [n = 165] vs 47.1% [n = 41], *P* < 0.001) or InSTI-based (44.5% [n = 93] vs 12.6% [n = 11], *P* < 0.001) rather than non-nucleoside reverse transcriptase inhibitor (NNRTI)-based (10% [n = 21] vs 43.7% [n = 38], *P* < 0.001).

We evaluated factors associated with early ART initiation by logistic regression analysis (Table 2). At multivariate analysis, higher plasma HIV-RNA load (adjusted odds ratio, aOR 1.76 per 1 log10 increase [95% confidence intervals, CI 1.29-2.40], *P* < 0.001) and more recent years of AEHI diagnosis (years 2012-2014 aOR 4.43 [95% CI 2.41-8.12] when compared to 2008-2011, *P* < 0.001) were independently associated with early ART initiation. This was after adjustment for symptomatic acute infection and CD4 lymphocyte number. Fiebig stage was not associated with early ART initiation.

**Table 2.** Factors associated with early ART initiation (logistic regression analysis)

	Univariate analysis OR (95% CI)	*P*	Multivariate analysis aOR (95% CI)	*P*
**Age, years (per 10 years increase)**	1.07 (0.84–1.36)	0.578		
**Male gender**	1.03 (0.50–2.14)	0.928		
**Risk factor:**				
**Heterosexual**	Ref.			
**Omo/bisexual**	1.41 (0.81–2.46)	0.224		
**IDU**	4.21 (0.50–35.27)	0.185		
**Other/unknown**	1.05 (0.42–2.63)	0.912		
**HBsAg+**	1.72 (0.35–8.29)	0.497		
**HCV coinfection**	0.80 (0.23–2.74)	0.725		
**Months from last negative HIV test, (per 1 month increase)**	1.01 (1.00–1.02)	0.157		
**Fiebig stage:**				
**I**	0.44 (0.14–1.46)	0.181		
**II**	1.27 (0.52–3.06)	0.595		
**III**	1.20 (0.54–2.69)	0.646		
**IV**	0.75 (0.40–1.39)	0.364		
**V**	Ref.			
**Symptomatic acute HIV infection**	2.38 (1.33–4.24)	**0.003**	1.97 (1.00–3.90)	0.051
**CDC stage C**	1.29 (0.13–12.60)	0.828		
**Concomitant STD**	0.99 (0.41–2.34)	0.977		
**Baseline laboratory results:**				
**CD4, cells/μL (per 100 cells/μL increase)**	0.90 (0.81–1.00)	**0.044**	0.92 (0.82–1.04)	0.921
**CD8, cells/μL (per 100 cells/μL increase)**	1.00 (0.98–1.03)	0.674		
**CD4/CD8 ≥ 1**	1.06 (0.42–2.64)	0.899		
**HIV-RNA, log10 copies/mL (per 1log10 copies/mL increase)**	1.88 (1.43–2.46)	**< 0.001**	1.76 (1.29–2.40)	**< 0.001**
**Year of diagnosis, 2012-2014 vs 2008–2011**	1.65 (1.42–1.92)	**< 0.001**	4.43 (2.41–8.12)	**< 0.001**

**Abbreviations:** IDU Intravenous Drug User; CDC Centers for Disease Control; STD Sexually Transmitted Diseases.

### Optimal Immunological Recovery

**Figure 1.**
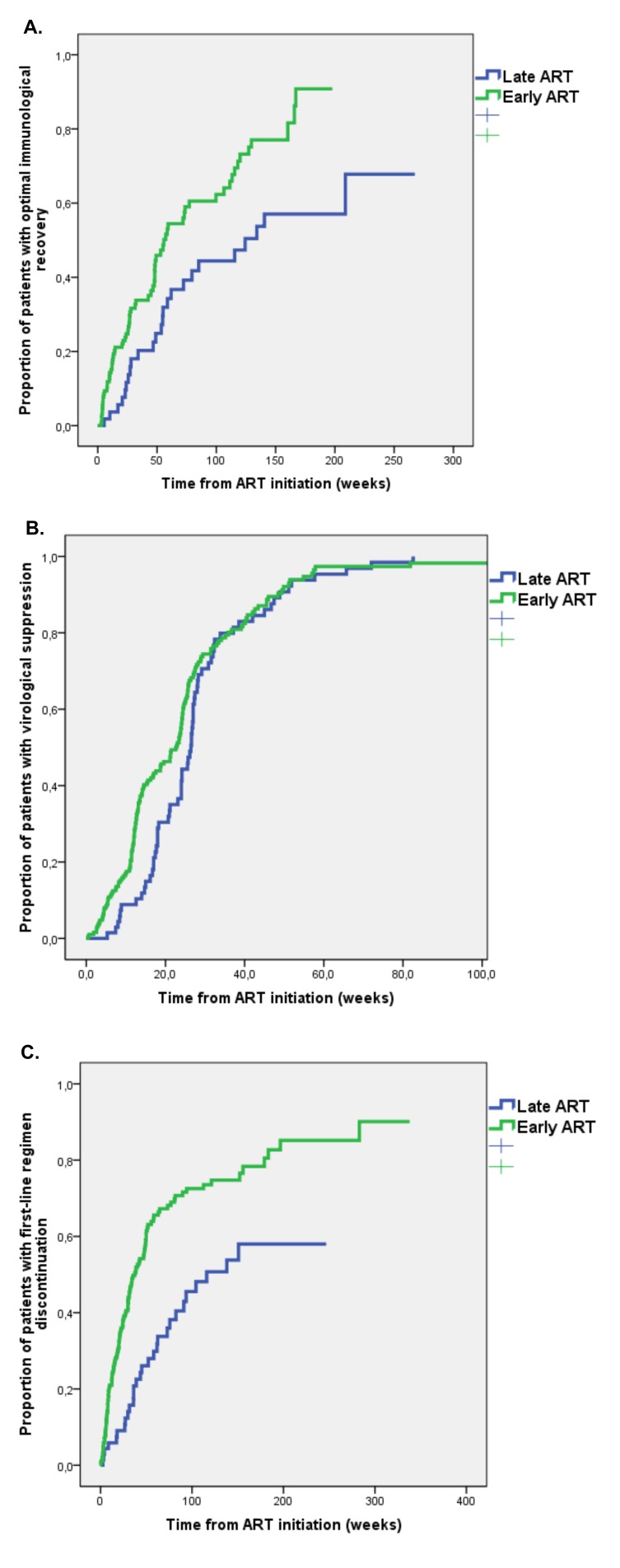
Kaplan Meier estimates of time to (A) optimal immunological recovery, (B) virological suppression and (C) first-line regimen discontinuation in Early versus Late ART group.

At the time of first-line regimen initiation, only 11 (3.4%) patients had optimal immunological status (ie, CD4 ≥ 500/µL plus CD4 ≥ 30% plus CD4/CD8 ratio ≥ 1). Of the remaining patients, 187 (58.3%) had follow-up immunological data. Optimal immunological recovery was achieved by 95 (50.8%) patients during a median follow-up of 35 weeks (IQR 15-85), with an incidence of 3.7 per 100 person-months of follow-up (PMFU). The Early ART group had a higher probability of optimal immunological recovery; at Kaplan Meier analysis the estimated incidence of optimal immunological recovery at 48 weeks was 39% in the Early ART group vs 22.5% in the Late ART group (*P* = 0.002 at log rank test) (Figure 1A).

We evaluated predictors of time to optimal immunological recovery by Cox regression analysis (Table 3). At multivariate analysis, early ART (HR 1.84 [95% CI 1.04-3.27], *P* = 0.036) confirmed an independent association with a higher probability of optimal immunological recovery after adjustment for Fiebig stage, the first ART regimen including > 3 drugs, calendar year, and baseline immunological status (ie, absolute and percentage CD4 count, CD4/CD8 ratio).

**Table 3.** Predictors of time to optimal immunological recovery defined as: CD4 ≥ 500cells/µL, CD4% ≥ 30% and CD4/CD8 ≥ 1 (Cox regression analysis) (n = 187)

	Univariate analysis HR (95% CI)	*P*	Multivariate analysis aHR (95% CI)	*P*
**Age, years (per 10 years increase)**	1.08 (0.88–1.33)	0.475		
**Male gender**	0.95 (0.53–1.71)	0.870		
**Risk factor:**				
**Heterosexual**	Ref.			
**Homo/bisexual**	0.97 (0.61–1.54)	0.899		
**IDU**	1.07 (0.37–3.08)	0.900		
**Other/unknown**	0.78 (0.37–1.67)	0.525		
**HBsAg+**	0.45 (0.11–1.82)	0.261		
**HCV coinfection**	0.74 (0.27–2.01)	0.551		
**Months from last negative HIV test, (per 1 month increase)**	1.00 (1.00–1.01)	0.300		
**Fiebig stage:**				
**I**	1.14 (0.27–4.92)	0.857	1.47 (0.33–6.63)	0.616
**II**	2.38 (1.26–4.49)	**0.008**	1.60 (0.82–3.12)	0.167
**III**	2.57 (1.42–4.65)	**0.002**	1.95 (0.97–3.90)	0.061
**IV**	2.07 (1.20–3.56)	**0.009**	1.44 (0.79–2.61)	0.236
**V**	Ref.			
**Symptomatic acute HIV infection**	1.13 (0.68–1.88)	0.650		
**CDC Stage C**	1.47 (0.20–10.61)	0.705		
**Concomitant STD**	1.68 (0.89–3.16)	0.107		
**Early (< 3 months from BL) ART initiation**	2.08 (1.30–3.32)	**0.002**	1.85 (1.04–3.27)	**0.036**
**First ART regimen including:**				
**NRTI**	1.81 (0.25–13.05)	0.557		
**NNRTI**	0.83 (0.51–1.34)	0.440		
**PI**	1.02 (0.65–1.59)	0.941		
**InSTI**	1.96 (1.20–3.21)	0.008		
**Entry inhibitor**	1.37 (0.78–2.42)	0.279		
**First ART regimen including > 3 drugs**	1.94 (1.23–3.05)	**0.004**	1.41 (0.84–2.37)	0.188
**Laboratory results at first ART initiation:**				
**CD4, cells/μL (per 100cell/μL increase)**	1.15 (1.06–1.24)	**0.001**	1.12 (1.02–1.23)	**0.020**
**CD4% (per 1% increase)**	1.06 (1.04–1.09)	**< 0.001**	1.04 (1.01–1.07)	**0.003**
**CD8, cells/μL (per 100cell/μL increase)**	0.98 (0.96–1.00)	0.053		
**CD4/CD8 ≥ 1**	4.15 (2.01–8.60)	**< 0.001**	2.84 (1.17–6.91)	**0.022**
**HIV-RNA, log10 copies/mL (per 1log10 copies/mL increase)**	1.15 (0.92–1.44)	0.225		
**Year of diagnosis, 2012-2014 vs 2008–2011**	1.24 (1.08–1.43)	**0.002**	1.56 (0.90–2.68)	0.112

**Abbreviations:** IDU Intravenous Drug User; CDC Centers for Disease Control; STD Sexually Transmitted Diseases; ART Antiretroviral Therapy; BL Baseline; NRTI Nucleoside Reverse Transcriptase Inhibitor; NNRTI Non-Nucleoside Reverse Transcriptase Inhibitors; PI Protease Inhibitor; InSTI Integrase Strand Transfer Inhibitor.

### Virological Suppression After ART Initiation

Follow-up virological data were available for 259/296 (87.5%) patients starting ART. During a median follow-up of 21 weeks (IQR 12-28), virological suppression was achieved by 219 (84.6%) patients with an incidence of 15.9 per 100 PMFU. By Kaplan-Meier analysis, the estimated proportion of patients achieving virological suppression after 48 weeks from ART initiation was 89.5%, with no significant differences between the Early ART (89.5%) and Late ART group (89.2%) (*P* = 0.159 at log rank test) (Figure 1B).

We evaluated predictors of time to virological suppression using Cox regression analysis (Table 4). At multivariate analysis, HBs-Ag positivity (adjusted hazard ratio, aHR 5.15 [95% CI 1.36-19.51], *P* = 0.016), CD4/CD8> 1 (aHR 4.05 [95% CI 2.012-8.16], *P* < 0.001), and HIV diagnosis in recent years vs years 2012-2014 (aHR 1.90 [95% CI 1.07-3.39]) in years 2012-2014 when compared to years 2008-2011, *P* = 0.028] were independently associated with a higher probability of virological suppression, while higher HIV-RNA (aHR 0.62 [95% CI 0.46-0.86] for each 1 log_10_ copies/mL increase, *P* = 0.003) was associated with a lower probability, after adjusting for early ART and time from last negative HIV test.

**Table 4.** Predictors of time to virological suppression – Cox regression analysis

	Univariate analysis HR (95% CI)	*P*	Multivariate analysis aHR (95% CI)	*P*
**Age, years (per 10 years increase)**	0.96 (0.85–1.09)	0.513		
**Male gender**	0.90 (0.62–1.31)	0.582		
**Risk factor:**				
**Heterosexual**	Ref.			
**Homo/bisexual**	1.08 (0.80–1.45)	0.634		
**IDU**	0.96 (0.46–2.02)	0.923		
**Other/unknown**	0.85 (0.52–1.39)	0.848		
**HBsAg+**	2.26 (1.06–4.84)	**0.036**	5.16 (1.36–19.51)	**0.016**
**HCV coinfection**	0.69 (0.35–1.35)	0.275		
**Months from last negative HIV test, (per 1 month increase)**	1.01 (1.00–1.01)	**0.009**	1.01 (1.00–1.01)	0.079
**Fiebig stage:**				
**I**	0.81 (0.41–1.64)	0.563		
**II**	1.46 (0.96–2.23)	0.077		
**III**	0.82 (0.55–1.24)	0.355		
**IV**	1.09 (0.78–1.51)	0.618		
**V**	Ref.			
**Symptomatic acute HIV infection**	1.09 (0.79–1.50)	0.612		
**CDC Stage C**	1.30 (0.41–4.08)	0.656		
**Concomitant STD**	0.76 (0.45–1.29)	0.311		
**Early (< 3 months from BL) ART initiation**	1.23 (0.92–1.65)	0.161		
**First ART regimen including:**				
**NRTI**	0.75 (0.24–2.36)	0.627		
**NNRTI**	0.79 (0.58–1.10)	0.160		
**PI**	1.06 (0.79–1.42)	0.716		
**InSTI**	1.22 (0.91–1.65)	0.187		
**Entry inhibitor**	0.94 (0.59–1.49)	0.799		
**First ART regimen including > 3 drugs**	1.04 (0.77–1.40)	0.807		
**Laboratory results at first ART initiation:**				
**CD4, cells/μL (per 100cell/μL increase)**	1.06 (1.00–1.13)	0.070		
**CD8, cells/μL (per 100cell/μL increase)**	0.99 (0.96–1.00)	0.165		
**CD4/CD8 ≥ 1**	2.31 (1.40–3.79)	**0.001**	4.05 (2.01–8.16)	**< 0.001**
**HIV-RNA, log10 copies/mL (per 1log10 copies/mL increase)**	0.76 (0.64–0.90)	**0.001**	0.63 (0.46–0.86)	**0.003**
**Year of diagnosis, 2012-2014 vs 2008–2011**	1.14 (1.05–1.23)	**0.002**	1.91 (1.07–3.39)	**0.028**

**Abbreviations:** IDU Intravenous Drug User; CDC Centers for Disease Control; STD Sexually Transmitted Diseases; ART Antiretroviral Therapy; BL Baseline; NRTI Nucleoside Reverse Transcriptase Inhibitor; NNRTI Non-Nucleoside Reverse Transcriptase Inhibitors; PI Protease Inhibitor; InSTI Integrase Strand Transfer Inhibitor.

### First Line ART Regimen Discontinuation

Follow-up data on antiretroviral regimens were available for 262/296 (88.5%) patients starting ART. During a median follow-up of 31 weeks (IQR 12-76), the first-line regimen was discontinued/switched by 148 (56.5%) patients with an incidence of 4.4 per 100 PMFU. Of 148 patients with first-line ART regimen discontinuation, 136 (91.9%) had modification of regimen composition and only 12 (8.1%) patients discontinued all ART (9 in the early and 3 in the late ART group). Early ART-treated patients had a higher probability of discontinuation of the first ART regimen; at Kaplan Meier analysis, the estimated incidence of ART discontinuation at 48 weeks was in 56.6% in the Early ART group versus 26.1% in the Late ART group (*P* < 0.001 at log rank test) (Figure 1C).

We analyzed predictors of time to first-line ART regimen discontinuation by Cox regression analysis (Table 5). At univariate analysis, patients starting early ART showed a higher risk of discontinuation (HR 2.34 [95% CI 1.55-3.52], *P* < 0.001), while those in Fiebig stages I-II showed a lower risk (HR 0.57 [95% CI 0.35-0.93], *P* = 0.024). However, at multivariate analysis, after adjustment for male gender, time from last negative HIV test, the first ART regimen including > 3 drugs, and calendar year, the association between early ART or Fiebig stage and regimen discontinuation was no longer demonstrated. The only independent predictor of first-line regimen discontinuation was initiating antiretroviral treatment with regimens including > 3 drugs (HR 1.88 [95% CI 1.13-3.13], *P* = 0.015).

**Table 5.** Predictors of time to first-line regimen discontinuation (Cox regression analysis) (n = 162)

	Univariate analysis HR (95% CI)	*P*	Multivariate analysis aHR (95% CI)	*P*
**Age, years (per 10 years increase)**	1.02 (0.88–1.18)	0.832		
**Male gender**	1.92 (1.15–3.20)	**0.013**	2.56 (0.92–7.09)	0.072
**Risk factor:**				
**Heterosexual**	Ref.			
**Homo/bisexual**	1.35 (0.93–1.97)	0.119		
**IDU**	2.54 (0.99–6.50)	0.053		
**Other/unknown**	1.55 (0.87–2.77)	0.142		
**HBsAg+**	0.80 (0.33–1.97)	0.627		
**HCV coinfection**	1.46 (0.68–3.12)	0.334		
**Months from last negative HIV test, (per 1 month increase)**	1.01 (1.00–1.01)	**0.049**	1.00 (1.00–1.01)	0.242
**Fiebig stage: I-II vs III-V**	0.57 (0.35–0.93)	**0.024**	0.66 (0.33–1.33)	0.246
**Symptomatic acute HIV infection**	1.03 (0.96–1.10)	0.486		
**CDC Stage C**	0.69 (0.17–2.90)	0.617		
**Concomitant STD**	0.98 (0.51–1.87)	0.947		
**Early (< 3 months from BL) ART initiation**	2.34 (1.55–3.52)	**< 0.001**	1.37 (0.72–2.62)	0.339
**First ART regimen including:**				
**NRTI**	0.86 (0.21–3.48)	0.830		
**NNRTI**	0.49 (0.31–0.76)	**0.002**		
**PI**	1.66 (1.12–2.47)	**0.012**		
**InSTI**	2.43 (1.71–3.45)	**< 0.001**		
**Entry inhibitor**	2.36 (1.50–3.73)	**< 0.001**		
**First ART regimen including > 3 drugs**	2.98 (2.09–4.25)	**< 0.001**	1.94 (1.16–3.25)	**0.012**
**Laboratory results at first ART initiation:**				
**CD4, cells/μL (per 100cell/μL increase)**	1.01 (0.93–1.10)	0.765		
**CD8, cells/μL (per 100cell/μL increase)**	0.99 (0.98–1.01)	0.402		
**CD4/CD8 ≥ 1**	0.84 (0.43–1.66)	0.624		
**HIV-RNA, log10 copies/mL (per 1log10 copies/mL increase)**	1.18 (0.97–1.43)	0.100		
**Year of diagnosis, 2012–2014 vs 2008-2011**	1.32 (1.18–1.48)	**< 0.001**	1.26 (0.72–2.20)	0.428

**Abbreviations:** IDU Intravenous Drug User; CDC Centers for Disease Control; STD Sexually Transmitted Diseases; ART Antiretroviral Therapy; BL Baseline; NRTI Nucleoside Reverse Transcriptase Inhibitor; NNRTI Non-Nucleoside Reverse Transcriptase Inhibitors; PI Protease Inhibitor; InSTI Integrase Strand Transfer Inhibitor.

## DISCUSSION

This is the first Italian multicentric study aimed to evaluate the use and the effect of early antiretroviral treatment of AEHI on a large observational cohort. The results of our study confirm that, during AEHI, early ART leads to enhanced rates of optimal immunological recovery compared to late ART, with no significant impact of this strategy on time to virological response or first-line regimen discontinuation. Conversely, Fiebig stage at diagnosis and the number of drugs included in the first ART regimen (intensified versus standard regimen) were not found to affect immuno-logical recovery. Moreover, our analysis allowed us to define predictors of virological response in the setting of AEHI. Pre-ART CD4+/CD8+ ratio, together with HIV replication levels, are independently associated with time to virological suppression, giving new insights into the interplay between immune function and virological control. The possible role of HBV-coinfection as a factor influencing a more rapid virological suppression needs to be clarified through prospective studies using standardized ART regimens in high endemicity settings.

In accordance with recent updates in international guidelines [23], in our real-life setting we confirmed a shift towards an early start of antiretroviral therapy during AEHI in recent years (2012-2014) compared to previous years (2008-2011), when nearly half of patients with AEHI started ART after 3 months from diagnosis. ART regimens prescribed as first-line treatment significantly differed between patients treated early or late after AEHI diagnosis; indeed, protease inhibitors and integrase inhibitors were more frequently used in the Early ART group, while NNRTI were mainly prescribed in the Late ART group. These differences in therapeutic choices can be explained by the more recent introduction of integrase inhibitors as the preferred first-line ART regimen in international guidelines and by the high genetic barrier of protease inhibitors allowing their use in early treatment of AEHI, while waiting results of genotypic resistance testing.

Despite the fact that data from clinical trials failed to demonstrate a clear clinical benefit of an intensified, multi-target ART as opposed to 3-drug standard regimens during AEHI [24-27], in our cohort we described a fairly frequent use of regimens including more than 3 drugs in early-treated AEHI patients (38.8% of subjects). Of note, in our cohort, early treatment was associated with greater risk of changes in ART regimen at univariate analysis, but patients who started early were also treated more frequently with regimens containing more than 3 drugs. Indeed, at multivariate analysis, after adjusting for the number of drugs in the first-line regimen, early treatment did not confirm an association with treatment discontinuation. Nevertheless, we recognized that a first ART regimen including more than 3 drugs was independently associated with a higher probability of first ART regimen discontinuation, thus reflecting the common approach to switch to simplified regimens after a first intensified phase, which led to viral suppression, or tolerability issues.

Early antiretroviral treatment in AEHI and a less compromised immunological status at ART initiation emerged as independent predictors of optimal immunological recovery (defined as reconstitution of absolute and percentage CD4 T-cell count, CD4/CD8 ratio), while Fiebig stage at diagnosis or administration of intensified regimens with more than 3 drugs did not show an effect on subsequent immunological outcome. To date, few studies have investigated the evolution of CD4/CD8 ratios in patients initiating ART close to infection. A sub-study [28] of the SPARTAC trial [29] investigating factors associated with time from ART initiation to CD4/CD8 ratio normalization, showed a higher probability of CD4/CD8 ratio normalization when ART was initiated closer to seroconversion (within 6 months from seroconversion). More recently, very early ART (initiated within 40 days of the estimated date of infection) in Fiebig I-II acutely infected patients was demonstrated to be associated with a significant increase in CD4/CD8 ratios [14], and the 1-year longitudinal evaluation of 83 patients starting ART within 120 days after the estimated date of infection displayed a better reconstitution of CD4/CD8 ratio compared to chronically infected subjects [15]. Here we confirmed that early ART, defined as ART introduced within 3 months from AEHI, has a beneficial effect on immune function in a large setting of patients, which were accurately defined according to Fiebig stage and thus excluded recent seroconverters, who were included in some of the previous analysis.

In our retrospective observational study, timing of ART initiation or types of antiretroviral drugs included in first-line regimens did not influence the achievement of virological suppression. Interestingly, we found that HBV coinfection, recent year of diagnosis, and a normal CD4/CD8 ratio at baseline (> 1) were independent predictors of time to virological suppression. On the other hand, as expected, higher HIV-RNA levels were associated with a longer time to virological suppression. The link between year of diagnosis and time to virological suppression could be explained by the recent wide use of antivirals able to rapidly reduce viral replication (ie, integrase inhibitors). This was recently observed in a similar study performed on acutely infected HIV patients who mostly began ART at their first medical appointment [30]. Similarly, patients with active HBV infection could have been treated with a tenofovir-based regimen more frequently than uninfected ones, thus masking a difference in the use of ART regimen. Indeed, much of the literature showed no difference between HIV monoinfected and HIV/HBV coinfected patients in achieving undetectable HIV-RNA after ART-initiation [31, 32], while recent observations reported a faster immune-reconstitution in HIV/HBV coinfected patients with high HBV-DNA viral load [33].

It is worth noting that we evidenced a role for baseline CD4/CD8 ratio as a predictor of time to virological suppression, suggesting that a preserved balance between T helper and T cytotoxic cells could provide a significant contribution in containing viral replication.

In the setting of untreated chronic HIV infection, CD4/CD8 ratio predicts time to AIDS development [34]. During treated HIV infection, expansion of CD8+ T cells (leading to low CD4/CD8 ratio) identifies a subgroup of individuals with a number of immunological abnormalities, namely an increased innate and adaptive immune activation, an immunosenescent phenotype, and a higher risk of morbidity/mortality [35]. In the setting of early acute HIV infection, an inverse correlation between HIV-1 DNA content and CD4/CD8 ratio was found, suggesting the potential use of normalized CD4/CD8 ratio as a valuable biomarker to identify individuals with a smaller size of HIV reservoir [36]. Moreover, some observations suggest that pre-ART CD4/CD8 ratio influences time to viral load rebound after analytical treatment interruption in acutely treated individuals [37, 38]. Our results showing a correlation between CD4/CD8 ratio pre-ART initiation and time to virological suppression suggest that T-cell immune exhaustion rapidly established after acute HIV infection can influence virological control. Further studies characterizing HIV-specific T cells, innate/adaptive activation, and immune exhaustion parameters in AEHI patients treated with standardized ART-regimens at different Fiebig stages could better define the real impact of these immunological abnormalities on virological control.

Our study, due to its observational nature, has several limitations. First, the availability of immunological data during follow-up was limited to less than 60% of our cohort, thus reducing the power of our population study. Second, it was not possible to adjust for unmeasured confounders, such as CMV infection, that recently emerged as an important predictor of CD4/CD8 ratio normalization [39]. Third, we lack data about HIV-DNA levels, thus we could not evaluate if timing or number of agents included in the ART regimen or immune parameters at baseline could influence the size of viral reservoir. A previous study [22] included the reduction of HIV-DNA in PBMCs in the definition of optimal viro-immunological recovery in ART-treated acutely infected patients, and the study demonstrated a higher probability of reaching optimal viro-immunological recovery in patients starting ART during AEHI compared with late ART, thus underlining a possible link between HIV-DNA limitation and immune-reconstitution during AEHI.

In conclusion, in a large observational setting, we observed that early ART during AEHI was able to enhance the achievement of optimal immune recovery in a significant way, which is rarely observed when ART is started during chronic infection, thus confirming the benefit of this strategy against the potential disadvantages of early continuous treatment. Moreover, we identified predictors of time to virological response in a well-characterized setting of AEHI, showing an independent role for CD4/CD8 ratio and giving insight into the research of new immunological markers to be considered in future algorithm selection of strategies for the cure of HIV.

## SUPPLEMENTARY DATA

**INACTION study group participating physicians and centers:**

Andrea Gori, Antonio Muscatello (Ospedale Maggiore Policlinico Milano); Nicola Squillace (Monza); Giuseppe Tambussi, Silvia Nozza, Marco Ripa, Raffele Dell'Acqua (H San Raffaele Milano); Andrea Antinori, Carmela Pinnetti (INMI Spallanzani Roma); Andrea Calcagno, Gianfranco Orofino, Ilaria De Benedetto, Micol Ferrara (Torino); Cristina Mussini, Vanni Borghi, Federica Carli (Modena); Benedetto Maurizio Celesia (Catania); Lucio Cosco, Carlo Torti (Catanzaro); Gabriella d'Ettorre (Umberto I Roma); Antonio Di Biagio (Genova); Emanuele Focà, Eugenia Quiros-Roland (Brescia); Antonina Franco (Siracusa); Diego Ripamonti, Franco Maggiolo (Bergamo); Roberto Gulminetti, Massimiliano Fabbiani (Pavia); Sandro Piga, Marzia Garau, Marco Campus (Cagliari); Stefano Rusconi, Tiziana Formenti, Sonia De Rose, Alessia Lai (H Sacco Milano); Giulia Marchetti, Camilla Tincati (H San Paolo Milano); Antonella Cingolani (H Gemelli Roma); Giordano Madeddu (Sassari).
